# Frequency of Migraine as a Chief Complaint in Otolaryngology Outpatient Practice

**DOI:** 10.1155/2015/173165

**Published:** 2015-01-28

**Authors:** Omar Sabra, Maria Muhammad Ali, Maha Al Zayer, Saleh Altuwaijri

**Affiliations:** ^1^Department of Otolaryngology, Saad Specialist Hospital, Prince Faisal Bin Fahd Road, P.O. Box 3250, Alkhobar 31952, Saudi Arabia; ^2^Clinical Research Laboratory, Saad Research and Development Centre, Saad Specialist Hospital, Prince Faisal Bin Fahd Road, P.O. Box 3250, Alkhobar 31952, Saudi Arabia

## Abstract

*Objective.* To identify the frequency of typical (headache and dizziness) and common atypical (ear fullness, pressure, pain, tinnitus, facial fullness, and nasal congestion) migraine symptoms as chief complaints among patients presenting to otolaryngology clinic.* Methods.* This is a descriptive study of prospectively collected data from a general otolaryngology practice. Typical migraine presentations were diagnosed by applying international headache society (IHS) criteria for migraine headache and Neuhauser's criteria for migrainous vertigo. Atypical otologic and rhinologic migraine symptoms were diagnosed using individualized criteria. Charts were reviewed at 6-month interval from the first presentation.* Results.* Out of 1002 consecutive patients, 10.8% presented with “migrainous chief complaint.” All migrainous chief complaint patients had a history of headache but not all of them presented with headache. Corrected female to male ratio in the migraine group was 3 to 1; age distributions were significantly different between the migraine and nonmigraine groups by applying *t*-test. Out of the atypical complaints, 86% of the patients had a history of concomitant typical presentation.* Conclusion*. Actual diagnostic criteria for migraine do not satisfy the diversity of its presentation. Investigating the history of migraine is enough to diagnose most atypical presentations. Sound knowledge about migraine seems essential for any ENT practitioner.

## 1. Introduction

Migraine is a common primary headache disorder. It is considered the most common cause of physician consultation for headache [1]. It is believed that half of the patients with migraine are undiagnosed. This might be in part due to the variability in its clinical presentation [2]. Headache and dizziness represent the only typical migraine manifestations in the head and neck area that are defined by clinical criteria (the IHS for headache and Neuhauser's for migrainous vertigo) [3, 4]. We know nowadays that migraine patients can as well present with multitude other head and neck symptoms. These can happen outside of the headache attack but still are believed to result from the same pathophysiologic migraine mechanisms. These atypical head and neck presentations of migraine are essentially related to the nose and the ear. The main nonheadache rhinologic symptoms include the following: facial and sinus pressure/fullness, frequently called “sinus headache,” nasal congestion, and less frequently runny nose [5, 6]. The main nondizziness ear symptoms include the following: ear fullness and pressure, ear pain, sound intolerance, and tinnitus [7, 8]. The majority of patients with these symptoms present to the otolaryngologist, who, if not experienced enough with the migraine symptoms, can easily miss the diagnosis and attribute those symptoms to primary ear or sinus disease without strong clinical evidence.

The objective of our study is to show the frequency of typical (headache and dizziness) and common atypical (ear fullness, pressure, pain, tinnitus, facial fullness, and nasal congestion) migraine symptoms as chief complaints among patients presenting or referred to otolaryngology clinics.

## 2. Methods

This is a descriptive study of prospectively collected data from a private otolaryngology practice in the eastern province of Saudi Arabia.

### 2.1. Patient Recruitment Criteria

1002 consecutive patients who presented or referred to the otolaryngology clinic over a 6-month time frame for any ENT chief complaint were included. Patients specifically presenting or referred for migraine management were excluded.

### 2.2. Diagnostic Tools

Typical migraine symptoms were diagnosed by their known criteria as follows.

Migraine headache was diagnosed using the international headache society (IHS) criteria for migraine with aura (IHS 1.1), migraine without aura (IHS 1.2), and probable migraine (IHS 1.6) [3].

Dizziness was diagnosed as migrainous when the patient met the Neuhauser criteria [4] for definite migrainous vertigo (DMV), while patients meeting the criteria of probable migrainous vertigo were considered migrainous only when showing response to migraine treatment.

For this study, atypical otologic and rhinologic migraine symptoms were diagnosed using individualized clinical criteria; (A) and (B) should be met to consider the migraine diagnosis:

- presence of at least one of the following:
  1. concomitance of the symptom to an IHS migraine headache,
  2. concomitance of the symptom to a Neuhauser's definite migrainous vertigo,
  3. response to a migraine preventive treatment;
- other primary or secondary causes ruled out.

To meet the second criterion a thorough history and a complete examination were performed on every patient before suggesting a migraine diagnosis. Paraclinical examinations were requested as needed according to the chief complaint as follows.

For ear fullness or pressure, pure tone audiometry and tympanometry were requested on all patients; only normal studies were included in the migraine diagnosis group of patients.

CT scan of temporal bones was requested to rule out a third mobile window syndrome.

For ear pain, a tympanometry and upper airway endoscopy were requested to rule out a primary or secondary cause of pain.

For tinnitus, audio and when unilateral, an MRI of brain was used to rule out other etiologies.

For rhinologic nasal symptoms, absence of purulence on endoscopy or sinus opacification on imaging and absence of nasal itching and sneezing were required to rule out sinusitis or rhinitis as an etiology.

The presence of nasal anatomical abnormalities including septal deviation, turbinate hypertrophy, and concha bullosa excluded a migraine etiology for nasal congestion when the laterality of the anatomical abnormality explains the laterality of the symptoms, while these anatomical findings were not considered as causes of headache [9].

## 3. Data Collection

Data collected included the chief complaint, its duration, and age and gender of patients in the migraine and nonmigraine groups. All migraine chief complaint patients were asked about history of headache.

Patients' medical charts in the migraine group were reviewed at 6 months interval from the first visit, to check for any change in the diagnosis during further follow up, and the duration of follow up of each patient during this 6 months interval. Response to migraine treatment was reassessed from chart review at 6 months interval after the first presentation to confirm the migraine diagnosis, when this was needed as a criterion. When response to treatment was not documented due to lack of follow up, suspected migraine individuals were removed out of the migraine group.

## 4. Data Analysis

The abovementioned chief complaints were classified as migrainous when meeting out proposed criteria upon first diagnosis and 6-month-follow-up; then the percentage of migraine chief complaint as well as its subcategories was calculated by the end of the study. Migraine and nonmigraine chief complaint groups were analyzed in gender and age distribution and duration of symptoms.

Student *t*-test was used to compare the age distribution of the migraine and nonmigrainous group of patient.

Percentage of typical and atypical migraine presentation was calculated as well as the percentage of patients with history of headache in the migraine symptoms group.

## 5. Results

Over 6-month period of time, 1002 patients presented consecutively with an ENT chief compliant. Out of these, 108 patients met one of the diagnostic categories for a “migrainous chief complaint,” and 894 were presenting with a “nonmigrainous chief complaint” including those with a suspected but not confirmed migraine diagnosis.

Overall in the whole group female to male ratio (F/M) was 441F/561M. In the “migrainous chief complaint” group of patients, there were 76 females and 32 males. When the female to male ratio in the migrainous chief complaint group was adjusted to the female to male ratio in the whole group, a female to male ratio of 3 to 1 was obtained, by applying the following simple formula.

Adjusted migraine (F/M) = (F/M) in the migraine group/(F/M) in the whole group.

**Figure 1:**
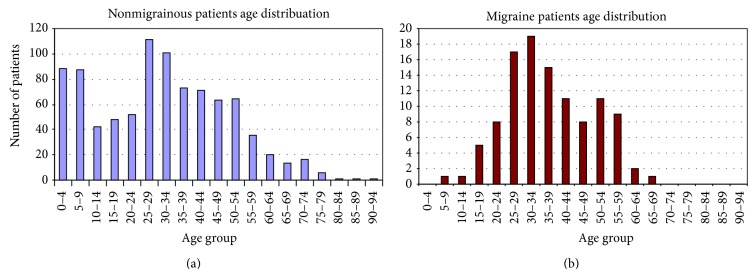
Age distribution charts for migrainous and nonmigrainous patients.

The age distribution for the nonmigrainous and migrainous chief complaints is presented in Figure 1.

Student *t*-test showed that age distribution was significantly different between the 2 groups with a *P* value of 0.00057; mean age for migraine was 37.1 ± 12.3 years, while for the nonmigrainous group mean age was 30.7 ± 18.9 years.

All of our migrainous chief complaint patients had a history of headache, but not all of them presented with headache. This headache history was tension type in 8 patients and was migraine in 100 patients out of which 67% had migraine without aura, 29% had migraine with aura, and 4% had probable migraine.

The distribution of diagnostic categories was as follows: 58 patients (53.7%) presented for a typical migraine presentation. Out of these typical patients 35 (32.4%) had migrainous vertigo and 23 (21.3%) had migraine headache. 50 patients (46.3%) presented for an atypical chief complaint out of which 34.2% were ear symptoms (mostly ear pain and fullness) and 14.8% nasal symptoms (mostly nasal congestion and sinus pressure) and 2.7% had ear and nose atypical chief complaint.

**Figure 2:**
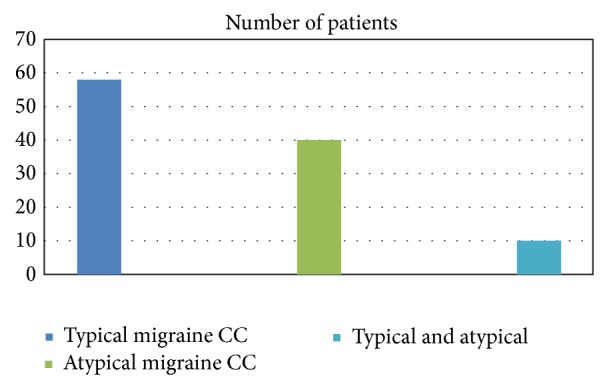
Percentage distribution of patients with typical and atypical migrainous chief complaints.

The type of atypical migraine chief complaints is shown in Figure 2.

**Figure 3:**
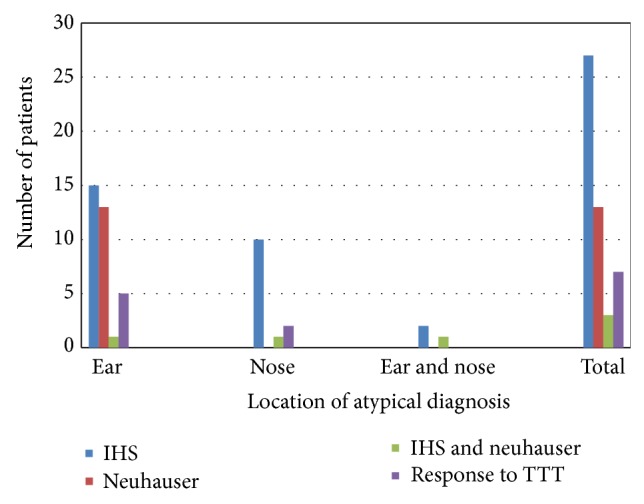
Distribution of atypical migraine patients according to the location of chief complaints and subcategories of criterion A.

The diagnostic criterion A was analyzed among our atypical migraine patients (concomitance to an IHS migraine headache, to DMV or response to migraine treatment); the diagnosis in 7 patients (14%) relied on response to migraine treatment, while 54% had a concomitant IHS migraine, 26% had concomitant DMV, and 6% had both concomitant IHS migraine headache and a DMV as shown in Figure 3.

The duration of the chief complaint ranged from 5 days to “lifelong.” 15 patients could not define the duration of symptoms in a clear number because of the chronicity. While the mean for the rest of the group was 33.3 months, only 17 patients defined the symptom duration as less than a month.

## 6. Discussion

Early description of migraine goes back to 1500 BC from Egypt [10]. Despite being known for a long time, no diagnostic test has been established for this disease and still clinical criteria are the only accepted diagnostic tool. In the field of headache, the IHS [3] has set criteria for the diagnosis of migraine while, in the field of dizziness, the Neuhauser criteria [4] are the most accepted criteria for the diagnosis of migrainous vertigo. In both fields, excluding other etiologies is a necessary criterion.

Otolaryngic manifestations of migraine include typical presentations of headache, especially when occurring in the sinus territory and dizziness. Both of these are objectively diagnosed using internationally accepted criteria (IHS criteria for headache and the Neuhauser for dizziness). Migraine manifestations include as well atypical otologic and rhinologic symptoms. For the sake of this study, and to have a consistent diagnostic way for any atypical migraine presentation, 2 conditions were set as essential criteria to define any atypical chief complaint as migrainous.

- First: the chief complaint should be concomitant to an IHS headache, to a Neuhauser DMV, or response to migraine preventive treatment.
- Second: other etiologies should be ruled out.

Using these criteria, 10.8% of the patients presenting to our clinic with an ENT complaint ended up with a migraine diagnosis; this is lower than the prevalence of migraine in the general population. One-year prevalence of migraine is estimated to be 11.7% [11]. In fact our study is not a prevalence study, but it counts only migraine symptoms as chief complaints in an ENT clinic. We believe a bigger percentage of our patients had migraine, but their presentation to the ENT clinic was for a nonmigrainous reason. This percentage of migrainous chief complaints is high enough to consider migraine as an essential part of any ENT practice.

Despite the fact that all of the migrainous chief complaints had a history of headache, only 21% had the headache as a chief complaint, while 35% presented with migrainous vertigo and around 46% presented with nonheadache nondizziness chief complaint. This can be explained by the fact that most patients do not consider the ENT clinic as a headache clinic; they usually present to us when they believe that their symptoms are related to a primary ear or nose pathology and that their headache is secondary to the ear or nose problem, while in reality a lot of these symptoms are secondary to the headache mechanism, not the opposite.

This study showed that 79% of migraine chief complaints in ENT were for nonheadache reasons; this should alert us, as otolaryngologists, to the importance of knowing nonheadache and atypical presentations of migraine. We believe that undiagnosed migraine [2] is in part related to the variability in presentation and that considering migraine in our differential might help in decreasing migraine underdiagnosis.

The criteria set in this study for atypical presentation restricted our migrainous group to the most evident cases as most of the patients were simply pulled out of the migraine group because they did not show up on followup. This led to incomplete documentation of response to treatment and inability to rule out other etiologies as investigations could not be completed. Probable migrainous vertigo is one category that was excluded from our statistics when no response to treatment was documented for the simple looseness of its criteria. It is a probable diagnosis by definition that might overlap with other dizziness diagnoses. As a result, we believe that migraine can be responsible of a bigger percentage of the ENT visits than the percentage calculated in this study.

Age and gender distribution in our migraine group were significantly different from the rest of patients' group. Distribution in the migraine group was very similar to the one in the general population with a female predominance that matches the known female to male ratio of 3 to 1 in the migraine general population [12]. The mean age of our migraine group (37.1 years) and the age of peak frequency (between 30 and 34) both were close to the peak of migraine one-year prevalence in the general population [13, 14].

However, this was not found in the nonmigraine group of patients, whose age distribution had 2 peaks of frequency; the first was in the range of 0 to 10 years, representing the pediatric ENT population, and the second was in the range of 25 to 34 years. The mean age in the nonmigraine group was 30.7 years.

86% of the patients diagnosed with atypical migraine symptoms according to the criterion A, proposed in our study, had concomitance of migraine headache according to IHS or DMV according to Neuhauser. As a conclusion, we propose that by applying the previously established criteria for migraine headache or DMV any otolaryngologists would be able to diagnose most of the atypical migrainous cases.

## 7. Migraine Symptoms in the ENT Literature

Migraine was considered a pure neurologic disease by majority of the physicians for a long time.

Although migraine is an associated risk factor for several otolaryngologic entities, we have hardly given any consideration in this regard or even tried to treat through this perspective.

Migrainous vertigo is one entity that was for a long time underdiagnosed by otolaryngologists and was rarely considered as a differential diagnosis for dizzy patients. In 1979 Slater first described benign recurrent vestibulopathy as an entity that showed to share common features of migraine [15]. Several years later, this same entity was highly correlated to migraine as 87% of those patients were found to be migrainous [16]. Migraine is also considered as a comorbid condition for BPPV, especially in its bilateral and idiopathic forms [17] and for Meniere's disease, more commonly encountered in its bilateral form as well [18]. These associations raise the question of possible undiagnosed migrainous vertigo patients in those studies. An interesting report about spontaneous perilymphatic fistula mentioned the presence of throbbing unilateral headache with light and sound sensitivity, usually occurring on the same side of the fistula [19]. This statement clearly describes a migraine headache picture and again reflects the underrecognition of migraine as a potential differential for some of these dizziness patients.

We assume the absence of an objective diagnostic test for a lot of dizziness entities to be the most probable reason for unrecognizing migrainous vertigo.

Neuhauser et al. [4], in 2001, made this entity more practical to be diagnosed by physicians previously inexperienced with migraine by establishing clinical criteria for diagnosing migrainous vertigo.

With the recognition of migrainous vertigo as an entity, some ear symptoms such as ear fullness and pain became evidently identified as migrainous just because of their association to migrainous vertigo [7, 8, 20]. In fact ear pain and fullness are the most common nondizziness migrainous ear symptoms. These are known to respond to the same migraine preventive treatment.

Another important differential for migraine related ear fullness and pain is Eustachian tube dysfunction. Migraine is known to be triggered by low barometric pressure, such as going to high altitudes or travelling in planes [21]. It has been observed that some of the patients present to the otolaryngologist on the assumption of having a Eustachian tube dysfunction. However, careful examination of those patients and tympanometry help in ruling out any Eustachian tube origin for these symptoms.

A key difference that sometimes might help differentiating both is the fact that Eustachian tube dysfunction is mainly symptomatic occurring while going to low altitudes such as during plane descent, whereas migraine gets triggered when going to high altitudes [22], although, in clinical practice, the difference might not be very obvious. Multiple other ear problems can cause ear fullness, but these are not usually triggered by altitudinal changes such as superior canal dehiscence or conductive hearing loss. Audiometry and appropriate imaging with a careful headache history would be helpful in differentiating migraine from other ear pathologies.

Rhinology is another field where migraine is frequently underdiagnosed. Headache in the sinus territory is frequently called by physicians and patients as sinus headache and is addressed automatically to a sinonasal etiology. In daily practice, otolaryngologists frequently associate headache to nasal anatomical problems such as turbinate hypertrophy, concha bullosa, a deviated nasal septum specially when associated with a contact mucosal point, and frequently to sinusitis. In fact, the 2004 classification of International Headache Society did not recognize chronic sinusitis as a valid cause of headache except in the setting of acute exacerbation. It was included as a potential cause of headache, but with strict criteria that proves causation according to the 2013 beta version of IHS classification [23]. The 2004 classification does not consider nasal anatomical problems such as septal deviation or turbinate hypertrophy as valid causes of headache and considers mucosal contact point headache as “of limited evidence.” This controversy on the rhinogenic cause of headache was addressed in a consensus between otolaryngologists, allergists, and neurologists but, again, the otolaryngologists could not show enough evidence in their publications to support the association between these entities and headache. Several publications have previously shown that the frequently called sinus headache is frequently a migraine headache. The new IHS classification describes migraine to be frequently exacerbated by sinus disease and should not be interpreted as only a sinusitis headache [23].

Several factors can lead to confusion in determining the etiology of symptoms whether sinonasal or migrainous, such as the triggerability of migraine by weather changes, usual coincidence of migraine and nasal allergy, and frequent presence of cranial autonomic symptoms such as nasal congestion and conjunctival injection and rhinorrhea during a migraine attack [5, 6, 24, 25].

Obstructive sleep apnea is one of the known triggers for several headache disorders including migraine [26]. Those patients frequently present to the ENT clinic looking for a nasal cause for their headache. Sleep apnea is a potential link between nasal blockage and migraine headache, as long as nasal blockage can worsen sleep apnea and possibly migraine headache.

Migraine was for a long time dealt with as a pure neurological chief complaint. The actual knowledge about migraine and the multitude of ENT symptoms has been made important for us as otolaryngologists in diagnosing the disease.

## 8. Conclusion

Migraine, despite being a highly prevalent headache disorder, is still an underdiagnosed clinical entity. We believe that the actual diagnostic criteria for migraine do not satisfy the diversity of its presentation. To achieve better success in identifying migraine patients, proficient knowledge about this disease is essential for otolaryngologists.

## Abbreviations

BPPV: Benign paroxysmal positional vertigo
DMV: Definite migrainous vertigo
CC: Chief complaint
IHS: International headache society.

## References

[1] Tepper S. J., Dahlöf C. G. H., Dowson A. (2004). Prevalence and diagnosis of migraine in patients consulting their physician with a complaint of headache: data from the landmark study. *Headache*.

[2] Pavone E., Banfi R., Vaiani M., Panconesi A. (2007). Patterns of triptans use: a study based on the records of a community pharmaceutical department. *Cephalalgia*.

[3] Headache Classification Subcommittee of the International Headache Society (2004). The international classification of headache disorders: 2nd edition. *Cephalalgia*.

[4] Neuhauser H., Leopold M., Von Brevern M., Arnold G., Lempert T. (2001). The interrelations of migraine, vertigo, and migrainous vertigo. *Neurology*.

[5] Lai T.-H., Fuh J.-L., Wang S.-J. (2009). Cranial autonomic symptoms in migraine: characteristics and comparison with cluster headache. *Journal of Neurology, Neurosurgery and Psychiatry*.

[6] Schreiber C. P., Hutchinson S., Webster C. J., Ames M., Richardson M. S., Powers C. (2004). Prevalence of migraine in patients with a history of self-reported or physician-diagnosed “sinus” headache. *Archives of Internal Medicine*.

[7] Neff B. A., Staab J. P., Eggers S. D. (2012). Auditory and vestibular symptoms and chronic subjective dizziness in patients with Ménière's disease, vestibular migraine, and Ménière's disease with concomitant vestibular migraine. *Otology and Neurotology*.

[8] Teixido M., Seymour P., Kung B., Lazar S., Sabra O. (2011). Otalgia associated with migraine. *Otology and Neurotology*.

[9] Levine H. L., Setzen M., Cady R. K. (2006). An otolaryngology, neurology, allergy, and primary care consensus on diagnosis and treatment of sinus headache. *Otolaryngology—Head and Neck Surgery*.

[10] Miller N., Newman N. J., Biousse V., Kerrison J. B. (2005). *Walsh and Hoyt's Clinical Neuro-Ophthalmology*.

[11] Lipton R. B., Bigal M. E., Diamond M., Freitag F., Reed M. L., Stewart W. F. (2007). Migraine prevalence, disease burden, and the need for preventive therapy. *Neurology*.

[12] Lipton R. B., Bigal M. E. (2005). The epidemiology of migraine. *The American Journal of Medicine*.

[13] MacGregor E. A., Rosenberg J. D., Kurth T. (2011). Sex-related differences in epidemiological and clinic-based headache studies. *Headache*.

[14] Lipton R. B., Stewart W. F., Diamond S., Diamond M. L., Reed M. (2001). Prevalence and burden of migraine in the United States: data from the American Migraine Study II. *Headache*.

[15] Slater R. (1979). Benign recurrent vertigo. *Journal of Neurology Neurosurgery and Psychiatry*.

[16] Cha Y.-H., Lee H., Santell L. S., Baloh R. W. (2009). Association of benign recurrent vertigo and migraine in 208 patients. *Cephalalgia*.

[17] Ishiyama A., Jacobson K. M., Baloh R. W. (2000). Migraine and benign positional vertigo. *Annals of Otology, Rhinology & Laryngology*.

[18] Radtke A., Lempert T., Gresty M. A., Brookes G. B., Bronstein A. M., Neuhauser H. (2002). Migraine and Ménière's disease: is there a link?. *Neurology*.

[19] Owen Black F., Pesznecker S. C., St. Jean J., Pensak M. L. (2001). Perilymph fistulae. *Controversies in Otolaryngology*.

[20] Johnson G. D. (1998). Medical management of migraine-related dizziness and vertigo. *The Laryngoscope*.

[21] Kimoto K., Aiba S., Takashima R. (2011). Influence of barometric pressure in patients with migraine headache. *Internal Medicine*.

[22] Silber E., Sonnenberg P., Collier D. J., Pollard A. J., Murdoch D. R., Goadsby P. J. (2003). Clinical features of headache at altitude: a prospective study. *Neurology*.

[23] Headache Classification Committee of the International Headache Society (IHS) (2013). The International Classification of Headache Disorders, 3rd edition (beta version). *Cephalalgia*.

[24] Eross E., Dodick D., Eross M. (2007). The sinus, allergy and migraine study (SAMS): CME. *Headache*.

[25] Saberi A., Nemati S., Shakib R. J., Kazemnejad E., Maleki M. (2012). Association between allergic rhinitis and migraine. *Journal of Research in Medical Sciences*.

[26] Rains J. C., Poceta J. S. (2012). Sleep-related headaches. *Neurologic Clinics*.

